# Addressing research capacity for health equity and the social determinants of health in three African countries: the INTREC programme

**DOI:** 10.3402/gha.v6i0.19668

**Published:** 2013-04-03

**Authors:** Karen Hofman, Yulia Blomstedt, Sheila Addei, Rose Kalage, Mandy Maredza, Osman Sankoh, Martin Bangha, Kathleen Kahn, Heiko Becher, Joke Haafkens, John Kinsman

**Affiliations:** 1 MRC/Wits Rural Public Health and Health Transitions Unit, University of Witwatersrand, School of Public Health, Johannesburg, South Africa; 2 Department of Public Health and Clinical Medicine, Umeå Centre for Global Health Research, Umeå University, Umeå, Sweden; 3 Dodowa Health Research Centre, Dodowa, Ghana; 4 Ifakara Health Institute, Dar-es-Salaam, Tanzania; 5 INDEPTH Network, Accra, Ghana; 6 Institute of Public Health, University of Heidelberg, Germany; 7 Amsterdam Institute of Advanced Labour Studies (AIAS), University of Amsterdam, Amsterdam, The Netherlands

**Keywords:** social determinants of health, health inequities, research, training, capacity building, e-learning, sub-Saharan Africa, Tanzania, South Africa, Ghana

## Abstract

**Background:**

The importance of tackling economic, social and health-related inequities is increasingly accepted as a core concern for the post-Millennium Development Goal framework. However, there is a global dearth of high-quality, policy-relevant and actionable data on inequities within populations, which means that development solutions seldom focus on the people who need them most. INTREC (INDEPTH Training and Research Centres of Excellence) was established with this concern in mind. It aims to provide training for researchers from the INDEPTH network on associations between health inequities, the social determinants of health (SDH), and health outcomes, and on presenting their findings in a usable form to policy makers.

**Objective:**

As part of a baseline situation analysis for INTREC, this paper assesses the current status of SDH training in three of the African INTREC countries – Ghana, Tanzania, and South Africa – as well as the gaps, barriers, and opportunities for training.

**Methods:**

SDH-related courses from the three countries were identified through personal knowledge of the researchers, supplemented by snowballing and online searches. Interviews were also conducted with, among others, academics engaged in SDH and public health training in order to provide context and complementary material. Information regarding access to the Internet, as a possible INTREC teaching medium, was gathered in each country through online searches.

**Results:**

SDH-relevant training is available, but 1) the number of places available for students is limited; 2) the training tends to be public-health-oriented rather than inclusive of the broader, multi-sectoral issues associated with SDH; and 3) insufficient funding places limitations on both students and on the training institutions themselves, thereby affecting participation and quality. We also identified rapidly expanding Internet connectivity in all three countries, which opens up opportunities for e-learning on SDH, though the current quality of the Internet services remains mixed.

**Conclusions:**

SDH training is currently in short supply, and there is a clear role for INTREC to contribute to the training of a critical mass of African researchers on the topic. This work will be accomplished most effectively by building on pre-existing networks, institutions, and methods.

In the discussions about building a post-Millennium Development Goal (MDG) framework in the years following their expiry in 2015, the importance of tackling economic, social, and health-related inequities is widely accepted as a core concern. Health inequities are defined by the World Health Organization (WHO) as ‘the unfair and avoidable differences in health status seen within and between countries’ (1), and the many voices calling for these disparities to be addressed include the UN Secretary General, a large number of UN member states, and a wide array of non-governmental organisations (NGOs) and development commentators (2–5).

The rationale for concern is simple. While the MDGs have effectively improved a number of national-level indicators in many countries, the aggregated data that are used to determine the overall status of these indicators do not shed any light on how things stand *within* a country. In other words, while a country as a whole may be doing well, particular groups of people – whether women, or the rural or urban poor – may actually be faring worse with regards to their access to water and sanitation, or to education, or to health care. Such inequities are demonstrably growing in many, if not most countries of the world (6–8).

One of the major, but perhaps less widely recognised lessons of the MDG experience has been the importance of building adequate in-country capacity to collect, analyse, and present policy-relevant data on the various targets. However, to date, ‘far too much of the growing amount of data cited in high-level reports is still based on poor-quality information, extrapolation, and guesswork’; and further, there are questions about the extent to which the data collection exercises have had a ‘positive and sustainable impact on local capacity’ (9, p. 38). The result is a global dearth of high-quality, policy-relevant, and actionable data that can help drive sustainable, equitable development. In particular, there are few data on inequities *within* populations, so that solutions are seldom focused on the people who need them most.

In 2008, the WHO's landmark Commission on Social Determinants of Health Report noted the central importance of monitoring, research, and training in order to address the social determinants of health (SDH) and health inequities (8). Greater focus on these areas is essential for decision makers to be able to identify geographical regions and/or groups of people among whom health inequities exist and where extra attention is therefore needed; and in the absence of good data, progress towards addressing the inequities will inevitably be compromised. In the expectation that health inequities and the social determinants of health will, one way or another, be included in the post-2015 development framework, it is all the more important that these technical areas are continually developed, so that high-quality, locally derived, and disaggregated data will be available to inform SDH intervention development, implementation, and evaluation.

To this end, an informed cadre of individuals who live and work in low- and middle-income countries (LMICs) is essential. These individuals will be able to disentangle the complex interaction between social determinants and health outcomes, and, further, they will be able to examine the whole development process in their own countries through a health lens. Above all, as citizens of the countries in question, the research findings that they produce will carry with them a degree of legitimacy in the minds of national-level decision makers that the findings of expatriate researchers may not achieve.

## INTREC

INTREC (INDEPTH Training and Research Centres of Excellence) was established with precisely these concerns in mind. The consortium consists of six institutions. Five of these are universities (Umeå University in Sweden; Gadjah Mada University in Indonesia; Heidelberg University in Germany; the University of Amsterdam in the Netherlands; and Harvard University in the USA), with the sixth being INDEPTH – the International Network for the Demographic Evaluation of Populations and Their Health in Low- and Middle-Income Countries. With its secretariat in Accra, Ghana, INDEPTH is an expanding global network, currently with 44 Health and Demographic Surveillance Systems (HDSSs) from 20 countries in Africa, Asia, and Oceania. Each HDSS conducts longitudinal health and demographic evaluation of rural and/or urban populations. INDEPTH aims to strengthen the capacity of HDSSs, and to mount multi-centre research to guide health priorities and policies in LMICs, based on up-to-date evidence (10). The topics of investigation in different centres include, among others, adult health and aging; non-communicable disease risk factor surveillance; migration, urbanisation, and health; studies on the epidemiology of epilepsy; and sexual and reproductive health.

INTREC was founded in response to one of the three ‘Principles of Action to achieve health equity’ identified by the SDH Commission Report. This included the need to ‘develop a workforce that is trained in the social determinants of health’, as well as to ‘measure the problem, evaluate action, [and] expand the knowledge base’ (8, p. 26). As a starting point, the INTREC team has conducted a situation analysis of SDH training needs among INDEPTH researchers (the demand side), which complements an analysis of existing SDH educational programmes in participating countries (the supply side). Based on the findings of this exercise, the INTREC programme aims to develop and provide relevant SDH-related training for INDEPTH researchers, thereby allowing the generation of new evidence on associations between social determinants and health. The programme also seeks to enable the sharing of this information through facilitating links between researchers and decision makers, as well as by ensuring that research findings are presented to decision makers in an actionable, policy-relevant manner. INTREC aims further to build the capacity of policy makers so that they will be interested to ask for and then use research findings. A number INDEPTH member centres are very well connected to policy makers – Navrongo HDSS in Ghana, for example, is an arm of the Ministry of Health. Their scientists collect a large quantity and variety of health-related data, but the all-important connection to social determinants is not often made. Through INDEPTH, therefore, INTREC offers a unique opportunity to provide decision makers across the global south with high-quality, timely research findings on SDH.

INTREC activities cover both Africa and Asia, with each continent served respectively by regional training centres in Ghana and Indonesia. These centres are focal points for research and training on the social determinants of health for the INTREC countries and, in due course, other LMICs. Thus, the centres in Ghana and Indonesia act to facilitate the development of South–South and North–South networking as well as training and research cooperation. See www.intrec.info for more details.

This paper focuses on three of the African INTREC countries: Ghana, Tanzania, and South Africa. These countries provide a good regional representation, by including West Africa, East Africa, and southern Africa. Further, these three countries are illustrative of a range of levels of inequity. Using Gini co-efficients as a measure of inequity – with a score of 0 representing total equity and a score of 1 representing extreme inequity – the three countries’ scores range from 0.67 for South Africa (the most unequal society in Africa) to 0.43 for Ghana, and 0.38 for Tanzania (11).

### Aims of the paper

This paper aims to provide an overview of the SDH-related training that is currently on offer in Ghana, South Africa, and Tanzania, including identifying where such training is available, as well as the gaps and barriers to training that currently exist. We also present material regarding current Internet capacity and access trends, as a basis for considering the extent to which future SDH courses may be offered through e-learning. Finally, we aim to raise awareness of the urgent need for developing a critical mass of well-trained SDH experts in LMICs, so that the issue of health equity can be properly addressed in the post-MDG world.

## Methods

One social scientist from each of the three countries (SA, RK, and MM) produced a report between February and September 2012, as a guide for the development of the wider INTREC programme. Each country report presented an SDH needs assessment, including material on the epidemiological and demographic background, the SDH policy landscape, SDH actors (NGO and government), and the SDH training opportunities available.

This paper draws mainly from the sections concerned with current SDH training opportunities. The methodology for these sections followed a similar approach in each of the countries, as explained below.

### SDH courses

We defined ‘SDH courses’ as those that included at least some discussion of issues that could be related to social determinants and/or inequities in health, and we actively searched for methodological, conceptual, and issue-specific courses. Since we did not know at the start which courses we would find on this basis, and since the nature of our search made some degree of subjective decision-making about choices almost inevitable, we were initially more inclusive rather than less so. In other words, courses were included on the principle that they could, under closer review, be subsequently excluded if we judged that they were only marginally associated with health inequities or social determinants.

The first step in identifying SDH-related courses in each country was to construct a list of all the Schools of Public Health: SDH is primarily taught on a post-graduate basis within the context of public health courses. The Schools were identified through personal knowledge supplemented by snowballing and online searches. Their respective websites were then visited, and the course and module outlines that were freely accessible online were then examined, searching for key terms such as ‘social determinants of health’, ‘health inequities’, and ‘determinants of disease’. In order to cast the net as widely as possible, we searched both for courses that are part of larger programmes – for example, Master of Public Health degrees – as well as for short courses and other stand-alone courses offered through Universities and NGOs. The rationale for including short and stand-alone courses was that they are likely to be the easiest way for health professionals in full-time employment to gain access to education about SDH. Where course outlines were not freely available online, course administrators were contacted with requests for details of each course and/or the course outline.

Departments outside Schools of Public Health that offered SDH-relevant courses were also identified using similar means. These departments included Sociology, Economics, Social Anthropology, Development and Social Sciences, and Biostatistics. All the courses that were listed on University websites were examined to see which were SDH-related. Where courses were not given on the website, teaching or academic staff were then approached and asked to provide the relevant information.

All of the information collected from this process was presented in tabular form, and a complementary narrative text was produced to highlight the main findings.

### Stakeholder interviews

A series of semi-structured interviews was conducted in each country with key stakeholders from a diversity of backgrounds, including the Ministry of Health, academics in universities and research institutes, Non-Governmental Organisations, SDH experts, and people from non-health sectors. Twelve interviews were conducted in Ghana, 11 in Tanzania, and seven in South Africa; although, in each country, there were potential respondents whom we approached but did not manage to interview, usually because of the time constraints they faced.

Ethical clearance was granted by the relevant authorities in each of the three countries for the interviews to be conducted. The interview guide covered a wide variety of topics, including, in relation to this paper, issues concerned with the training of researchers in SDH, and SDH training gaps that INTREC could fill. The interview data were subjected to thematic analysis.

### Internet availability

In order to assess the feasibility of opportunities for SDH education through e-learning, information regarding Internet access and penetration was gathered in each country through online searches. The searches sought to establish the proportion of people with access to the Internet, trends in the extent and quality of service provision, as well as the reliability and speed of the connections.

## Results

The material collected suggests that SDH-relevant training is available in Ghana, Tanzania, and South Africa, but 1) the number of places available for students is limited; 2) the training tends to be public-health-oriented (since it is usually taught in Schools of Public Health) rather than inclusive of the broader, multi-sectoral issues associated with SDH; and 3) insufficient funding places limitations on both students – who have to self-fund or obtain funding themselves – and on the training institutions themselves, thus affecting participation and quality. We also identified rapidly expanding Internet connectivity in all three countries, though the current quality of the services remains mixed.

Details regarding each of these points are given below. Names of the training institutions are not provided in order to preserve anonymity. Since each of the points appeared to some extent or another in the data for each of the three countries, it was not seen as necessary to identify the individual sources here.

### Limited availability of training

Most SDH-related training in all three countries takes place in Master of Public Health (MPH) programmes within Schools of Public Health. The availability of training varies considerably: there are nine Schools of Public Health in South Africa, three in Tanzania, and one in Ghana. The courses that we considered to be SDH-relevant included, for example, Social Epidemiology, Public Health and Society, and Women's Health in Sub-Saharan Africa. However, a number of these courses are given as electives rather than core courses, meaning that some MPH graduates may not be exposed to or have a good grasp of them.

Some courses are offered outside Schools of Public Health as part of other postgraduate curricular, or as stand-alone short courses. These include topics ranging from the Sociology of Health to HIV/AIDS and Society, and Nutritional Security for Health and Development. Not surprisingly, SDH training is rare outside the health sector. While education is highlighted as a critical social determinant of health, few, if any, educational training courses in our three countries cover SDH.

Overall, while there is a good diversity in the SDH-relevant courses given in the three countries, the actual number of courses available for each country is quite limited. Consequently, entry into the various programmes in each of the three countries is highly competitive, and many people who would like to receive training in SDH are unable do so. In one institution, for example, we learned that 298 applications for the 2011/2012 MPH course had been received, but only 88 students could be admitted (30% of those who applied).

See Annex 1 in each of the Ghana, South Africa, and Tanzania country reports for full details of the SDH-related courses identified (http://www.intrec.info/countryreports.html).

### Insufficient SDH-specific training

Although much of the SDH-related training takes place at schools of public health, several interview respondents felt that the specifics of SDH were not adequately covered in the public health training curricular, and that these courses were not practical or detailed enough to equip students with a clear conceptual grasp of SDH. For example, while various aspects of their courses may deal with inequities and other SDH-related issues, SDH is not the focus of the training and the link is often not made explicit. As one informant said, her students were therefore unprepared to address ‘real issues’ when they finished their courses.

A further training gap is to be found in the relatively limited focus given in the various curricular to research methods (qualitative and quantitative), and to health economics. Both these areas are critical to SDH, insofar as they provide the basis for showing evidence (or not) of intervention effectiveness and its relationship to equity; how and why an intervention might be improved; and, critically for policy makers, evidence (or not) of cost effectiveness for different sub-populations.

### Staffing, funding, and institutional infrastructure

The issues of staffing, funding, and institutional infrastructure are intertwined, and when any of them are less than adequate, bottlenecks in the provision of education are almost inevitable. At one of the institutions we surveyed, the staff reported feeling under-manned and overworked. Furthermore, their remuneration was not seen as competitive, which meant that remaining motivated was difficult, as was recruiting high-quality new staff to reduce their burden.

We also learned of one case of a promising course on Social Epidemiology that was, in the end, dropped, due to insufficient funds. On a more hopeful note, reference was made to an SDH-related portion of an MPH programme whose financial security is being covered, at least for the time being, by USAID funding. While this particular financing remains quite insecure, it does nonetheless indicate recognition by the donor community of the need for such support.

As for the students themselves, few Schools of Public Health offer scholarships for their MPH programmes, which means that students need to secure their own funding. Most students are supported by their employers or by other funding agencies, but it is clearly essential that for SDH training to be accessible in such institutions, it must be made as inexpensive to students as possible in order to give those without access to substantial resources the opportunity to participate.

### Access to the Internet

Geographical distance between course venue and potential students is one of the major constraints facing people who want to take classroom-based courses. E-learning offers a way around this challenge, but it is important to understand the extent and quality of Internet penetration into different settings before Internet-based educational opportunities can be developed.

Although the details differ in each of the three countries, the overall picture with regards to Internet availability is one of rapidly increasing access (particularly into rural and previously under-served areas), falling prices, and improving services. These improvements are the result of 1) a series of high-capacity submarine cables laid in recent years that carry Internet traffic between various African countries (including the three countries discussed here) and the rest of the world, and 2) substantial private investment supported by government policies that have boosted access within each of the countries.

That said, there is still a long way to go before anything approaching universal access is reached. Internet penetration at the end of 2011 has been estimated at 8.4% of the population in Ghana, 11.5% in Tanzania, and 13.9% in South Africa (12). Furthermore, some of the Internet services on offer can be unreliable, with regular disconnections occurring and/or slow or limited connectivity in some areas. In spite of considerable investment by many INDEPTH member centres in upgrading their Internet services, their connections are still not as reliable or fast as many would like, and the cost of increasing broadband access further remains prohibitive.

Currently, nearly all the SDH-related courses available in Ghana, Tanzania, and South Africa are given on a face-to-face, classroom basis. With the ever-expanding availability of good quality Internet services in all three countries, however, online education on SDH will increasingly be a viable, and, perhaps, less inexpensive means of meeting the SDH training demand. Significant investment will be required to transform course modules into online courses and so enable more students to receive the training. It should be stressed, however, that Internet-based training is unlikely to completely replace classroom-based education, which will continue, in one form or another, to play an important role.

## Discussion

### SDH training is in short supply

The measurement of social determinants of health and knowing how to address them will be especially critical in the post-MDG world. Addressing poverty and inequities will need a stream of trained professionals who can understand and tackle the issues found in different economic sectors, but who are focused on this work through a prism of health.

Our analysis shows the range of SDH-related programmes and courses that exists in three Anglophone countries, in East, West, and southern Africa. The majority of the programmes are in South Africa, but in none of the three countries are programmes either focused specifically on SDH, and neither are they available as online courses – although some universities are currently developing the infrastructure needed to provide e-learning.

We are not calling for a wholesale shift in the conceptual basis of public health training, but a process towards mainstreaming SDH into the curriculum is clearly necessary within the post-2015 paradigm. Students of public health and other related areas need to develop a firm understanding of the concept and practical realities of SDH, so that these become central to their professional thinking. To this end, new, SDH-related courses are required, both classroom-based and online. These might deal with, for example, the legislative and fiscal policies required to bolster efforts to reduce the burden of non-communicable diseases; or urban health in children; or the promotion and maintenance of a healthy workforce. Most of these courses will likely be provided through existing institutions of higher education, but INTREC will also contribute within a very particular niche.

Our call for mainstreaming SDH training within public health training complements a related call made recently to mainstream teaching of sexual and reproductive health and rights (SRHR) (13). SRHR themselves constitute major social determinants of health and hence it may be mutually beneficial for proponents of both SRHR and SDH to work together strategically in the promotion of their respective agendas.

### Building on what already exists

The work of INTREC revolves around INDEPTH's unique global scientific network. Researchers from the network's 44 member centres in 20 countries routinely collect detailed and robust longitudinal demographic and epidemiological data in order to understand the changes taking place in their populations. Since these datasets tend to include both health outcomes and variables related one way or another to the social determinants of health, they offer great potential for investigating SDH, and, thereby, for providing powerful advocacy material to convince policy makers and donors to invest in addressing the major social determinants that adversely affect health.

However, to date, the link between the social determinants of health and health outcomes has not been made as often and as explicitly as it could be by many of the INDEPTH member HDSS centres. This may be due to insufficient conceptual understanding of SDH and health inequities among the scientists, as well as a related shortage of technical capacity on the subject. Since a core objective of INTREC is to provide such knowledge and technical capacity, tailored to the particular conditions and capacities to be found among INDEPTH scientists, we are aiming to build an important additional component onto the work already being undertaken by INDEPTH.

With European Union support, INTREC will directly contribute to one of the Commission on Social Determinant's core recommendations, by helping to ‘develop a workforce that is trained in the social determinants of health’ (8). But INTREC is not alone in this effort – there are a few other groups that have begun to work towards promoting training in SDH in Africa. One of these is the Consortium for Advanced Research Training in Africa (CARTA), an initiative of nine African Universities, four African research institutes and select northern partners. The goal of CARTA – which is supported by a number of high profile funders, including the Wellcome Trust, the Carnegie Corporation of New York, the Bill and Melinda Gates Foundation, the Ford Foundation, and the Department for International Development – is to strengthen the capacity of African institutions to conduct and lead internationally competitive research that enhances the health and development of African populations. An important component of this is an innovative model for doctoral training in sub-Saharan Africa that prepares its graduates to better address questions of contemporary policy relevance, such as the social determinants of health (14). CARTA fellows are currently junior faculty of the nine participating institutions, so ensuring on-going capacity development within these institutions. See www.cartafrica.org.

It would also be appropriate to seek synergies between INTREC and existing Masters level programmes and free-standing short courses, thereby further expanding the stream of SDH-trained professionals. One such opportunity could be through the INDEPTH Leadership Programme, which runs a 2-year MSc programme in Population-based Field Epidemiology, developed in collaboration with the University of the Witwatersrand, Johannesburg, South Africa. The programme includes time spent at three learning sites, in Navrongo (Ghana), the Africa Centre (South Africa), and Ifakara (Tanzania) HDSS centres. The inclusion of an SDH component in this programme would make an important additional contribution to expanding SDH-related capacity among INDEPTH scientists.

We recognise that bringing new courses into established, busy postgraduate training curricula – and especially new courses at the ‘softer’ end of public health, such as those on SDH – will be challenging. However, since people who are also closely engaged with INTREC run CARTA and the INDEPTH Leadership Programme, we anticipate that it may be feasible to facilitate integration of our courses into these programmes. Further, by demonstrating the health and financial gains to be had at national level through addressing social determinants, we are also of the view that parallel, context-specific research on SDH will help to convince the curriculum designers of other programmes of the importance of SDH for their own courses.

Technically, courses based on a mix of printed and electronic training have been successfully run by the African Medical and Research Foundation (AMREF) in Kenya, where conditions are similar to those found in many of the INTREC countries. AMREF has developed a series of computer-based training modules that are being delivered through more than 100 e-learning centres, including to some of the most remote areas of the country. To date, more than 4,500 nurses have enrolled in the programme, using both print and e-learning modules (15). This mixed methods training model is therefore demonstrably feasible in Africa, and should be seen as a viable option for INTREC training.

## Conclusion

A central pillar of the global effort to address health inequities, as identified by the WHO's Commission on Social Determinants of Health, is to ‘Measure and understand the problem and assess the impact of action’ (1). An array of resources will be required to advance a training agenda that will produce the human and institutional capacity needed to support this work. These resources will have to come from forward-thinking donors, foundations, research funders, and international aid agencies, who will need to commit to the agenda in order to ensure that scholarships can be obtained and that courses are properly supported. Local buy-in will also be required from national-level political leaders and from the academic leadership at Schools of Public Health. Further, institutions of higher learning will need to show flexibility by permitting or even encouraging cross-faculty training in SDH-related issues. We hope that this paper contributes to increasing awareness of the urgent need to develop these all-important financial and political resources.

For its part, INTREC will begin by contributing to the training of a critical mass of African researchers who understand how to adopt health indicators to measure progress and achievements in sustainable development. Through the unique global network offered via INDEPTH, this work aims to enhance the health of the 44 communities under study by the HDSS centres as well that of the wider populations in the 20 countries where the centres are based. In addition, on the premise that the whole is much greater than the sum of its parts, we expect that INTREC will contribute substantially to the global movement that aims to address health inequities and the social determinants of health.
